# Altered white matter connectivity is linked to language abilities in children with autism spectrum disorder: An automated fiber quantification study

**DOI:** 10.3389/fpsyt.2025.1731647

**Published:** 2026-01-20

**Authors:** Aiwen Yi, Kaiyu Huang, Yubin Hu, Shuiqun Zhang, Qingshan Huang, Yaqiong Xiao

**Affiliations:** 1 Guangdong Women and Children Hospital, Guangzhou, China; 2 Department of Psychosis Studies, Institute of Psychiatry, Psychology and Neuroscience, King’s College London, London, United Kingdom; 3 Faculty of Life and Health Sciences, Shenzhen University of Advanced Technology, Shenzhen, China; 4 Department of Pediatrics, The Third Affiliated Hospital of Guangzhou Medical University, Guangzhou, China; 5 Guangdong Provincial Key Laboratory of Major Obstetric Diseases, Guangzhou, China; 6 Guangdong Provincial Clinical Research Center for Obstetrics and Gynecology, Guangzhou, China; 7 Guangdong-Hong Kong-Macao Greater Bay Area Higher Education Laboratory of Maternal-Fetal Joint Medicine, Guangzhou, China; 8 Foshan Clinical Medical School of Guangzhou University of Chinese Medicine, Foshan, China

**Keywords:** autism spectrum disorder, automated fiber quantification, diffusion tensor imaging, language deficits, language-related tracts, white matter connectivity

## Abstract

**Introduction:**

Recent studies using Automated Fiber Quantification (AFQ) have revealed localized white matter connectivity alterations in individuals with autism spectrum disorder (ASD), offering insights beyond traditional tract-wise Diffusion Tensor Imaging (DTI) analyses. However, the relationship between these alterations and language variability in preschool-aged children with ASD remains poorly understood.

**Methods:**

This study included 28 children with ASD and 22 typically developing (TD) peers aged 1.5–6.07 years. Using AFQ, we examined eight language-related tracts—bilateral arcuate fasciculus, inferior fronto-occipital fasciculus, inferior longitudinal fasciculus, and superior longitudinal fasciculus—at both tract-wise and point-wise levels. We analyzed the white matter alterations in metrics including fractional anisotropy, mean diffusivity, radial diffusivity, and axial diffusivity, and correlated these metrics with language abilities and ASD symptom severity.

**Results:**

Both groups exhibited significant lateralization patterns, though no between-group differences in lateralization were found. However, ASD and TD groups showed distinct associations between white matter lateralization and language abilities. Tract-wise comparisons revealed no significant group differences, but point-wise analyses identified localized alterations in DTI metrics within the ASD group. While these alterations showed different patterns of association with language abilities in the ASD and TD groups, the between-group comparison of these association patterns did not reach statistical significance. Additionally, DTI metrics correlated significantly with ASD symptom severity.

**Discussion:**

Our findings underscore the importance of white matter lateralization and microstructural integrity in supporting language abilities in young children with ASD. The study provides novel insights into the neuroanatomical foundations of language deficits and their association with symptom severity, highlighting the value of point-wise analyses in understanding ASD-related connectivity alterations.

## Introduction

Autism Spectrum Disorder (ASD) is a complex neurodevelopmental condition marked by challenges in social interaction, communication, and repetitive behaviors. While these behavioral characteristics have been extensively documented, the neural mechanisms underlying ASD remain less understood, especially during early childhood—a critical period for brain development (1). Understanding these mechanisms is vital not only for identifying early biomarkers but also for informing intervention strategies that could potentially alter the developmental trajectory of affected children (2).

In recent years, neuroimaging techniques have provided a window into the brain’s structure and function in ASD, with diffusion tensor imaging (DTI) emerging as a particularly valuable tool. DTI allows researchers to explore the microstructure of white matter, the brain’s connective tissue that facilitates communication between different regions. White matter integrity is crucial for efficient neural communication, and disruptions in these pathways may contribute to the core symptoms of ASD, and alterations in these tracts have been proposed as a potential neural substrate for the cognitive and behavioral symptoms observed in ASD (3–7).

Previous DTI studies have reported alterations in various white matter pathways in individuals with ASD. For example, an DTI study found greater fractional anisotropy (FA)—a measure of white matter integrity—in toddlers with ASD across multiple tracts, including fronto-frontal, fronto-temporal, fronto-striatal, fronto-amygdala pathways, as compared to TD toddlers (8). Some studies have also identified differences in white matter tracts involving in language processing, such as the arcuate fasciculus (AF), inferior longitudinal fasciculus (ILF), superior longitudinal fasciculus (SLF), and inferior fronto-occipital fasciculus (IFOF) (e.g., 9–11). These tracts connect brain regions responsible for language comprehension, production, and social communication—functions often impaired in individuals with ASD.

Despite a growing body of research, findings on white matter alterations in ASD have been inconsistent. While some studies have reported reduced FA in children with ASD, suggesting disrupted white matter integrity (3, 12, 13), others found no significant differences or even increased FA in specific tracts (13–15). These discrepancies may be due to differences in sample characteristics, methodologies, or the specific tracts examined. Additionally, the choice of tracts and the statistical methods used to analyze DTI data can also contribute to the variability. As such, more research is needed to clarify white matter alterations in ASD, particularly in young children, where early detection and intervention are critical.

In addition to traditional DTI analyses, recent studies have explored the alterations in white matter connectivity in ASD using a newly developed approach, i.e., Automated Fiber Quantification (AFQ) (16, 17). This approach allows for a detailed assessment of white matter by measuring diffusion metrics along the entire length of fiber bundles, rather than averaging them over an entire tract. The AFQ can reveal localized changes in specific tracts that might be overlooked in conventional DTI analyses (18–20). This method has been increasingly applied to investigate tract-specific alterations in children with ASD (18, 20, 21). For example, Li et al. (18) used AFQ to analyze multi-site datasets and found altered white matter connectivity in the ventral language networks in individuals with ASD. They observed reduced FA and increased radial diffusivity (RD) in certain segments of the AF and ILF, highlighting localized disruptions in white matter that may contribute to language impairments in ASD. Such findings underscore the potential of AFQ to reveal subtle, region-specific white matter changes that could help the heterogeneous nature of ASD, especially in young children—a topic that remains understudied.

In the present study, we aimed to investigate early white matter alterations that may contribute to the pathophysiology of language deficits in children with ASD. We enrolled young children aged 1.5–6 years with ASD and typically developing (TD) controls and used AFQ to examine key DTI metrics—FA, mean diffusivity (MD), axial diffusivity (AD), and RD—across language-related white matter tracts, including the AF, IFOF, SLF, and ILF. These metrics provide important insights into white matter integrity: FA reflects fiber density and myelination, MD indicates overall tissue diffusivity, and AD and RD provide information about axonal integrity and myelination, respectively. We first examined lateralization patterns in key white matter tracts for both ASD and TD groups, expecting different lateralization patterns in these two groups. Next, we explored the relationships between the lateralization index (LI) and language abilities within each group, hypothesizing different association patterns. Furthermore, we compared DTI metrics at both the tract-wise and point-wise levels, predicting that ASD children would exhibit significant alterations in white matter integrity compared to TD children. Additionally, we investigated the associations between DTI metrics and language abilities at both the tract-wise and point-wise levels within each group, anticipating distinct association patterns in the ASD group. Finally, given the interplay between language deficits and symptom severity in ASD (22), we examined relationships between integrity of language-related white matter tracts and the severity of ASD symptoms, expecting that significant associations would emerge between these variables, thus providing further insight into the neuroanatomical basis underlying the association between language deficits and symptom severity in ASD.

## Materials and methods

### Participants

The current study enrolled children aged 1.5–6 years. A total of 50 participants aged 1.5–6.07 years (mean age: 3.12 ± 1.28 years) were included in the analysis, all recruited from Foshan Fosun Chancheng Hospital, China, between November 2021 and May 2023. All participants were assessed using the Gesell Developmental Schedules (GDS), developmental domains including fine motor, gross motor, personal-social, language, and adaptive behavior (23). Their parents or guardians completed the Autism Behavior Checklist (ABC) (24), a standardized questionnaire for assessing autistic behaviors and symptom severity. Children in the ASD group (n = 28; mean age: 3.24 ± 1.26; age range: 1.67–6.07 years) met the DSM-V criteria for ASD following a clinical interview and were assessed using the Autism Diagnostic Observation Schedule (ADOS Module 1 or 2) (25), administered by the same clinician. In addition, all children with ASD had ABC scores ≥ 53. Children in the TD group (n = 22; mean age: 2.97 ± 1.31 years; age range: 1.5–5.81 years) had GDS total scores > 86 and ABC scores < 47, indicating normal development and the absence of ASD. All children were native Mandarin and Cantonese speakers with normal hearing and without family history of mental or psychiatric disorders, including no family history of autism spectrum disorder.

Written informed consent was obtained from the parents or legal guardians of all participants in accordance with the Declaration of Helsinki. This study was approved by the Institutional Review Board of Foshan Chancheng Hospital (No. IRB-ATT-002-61).

### Data acquisition

To ensure that participants remained asleep during the MRI scan, oral chloral hydrate was administered for sedation at a dose of 0.5 mL/kg, with a maximum dose of 10 mL (at a 0.5% concentration) before scanning. All sedated children were continuously monitored by trained medical staff throughout the scan to ensure the safety of the participants.

DTI data were acquired from all participants using a 3.0 T SIEMENS Skyra scanner at Foshan Fosun Chancheng Hospital, China, with the following parameters: TR = 6800 ms, TE = 92 ms, slice thickness = 2 mm, matrix size = 128 × 128 mm, FOV = 220 × 220 mm², flip angle = 90°, gap = 0.2 mm, voxel size = 1.7 × 1.7 × 2 mm³, 60 directions, b-value = 1000 s/mm², 12 b0 image (b = 0 s/mm^2^), 56 slices, 72 volumes. The total scan duration was approximately 15 minutes.

### Data preprocessing and tract quantification

Prior to data analysis, all DTI scans were manually inspected for artifacts and overall image quality. Because all scans were acquired under sedation, motion-related artifacts were minimal, and no scans were excluded based on quality concerns. The diffusion-weighted imaging data were processed using the Python Automated Fiber Quantification (pyAFQ; https://yeatmanlab.github.io/pyAFQ/; 16), an open-source tool that facilitates customized analysis. The “ParticipantAFQ” function was used to handle the entire workflow, including tractography, registration, segmentation, cleaning, profiling, and visualization. Preprocessing steps such as motion correction, eddy current correction, and tensor estimation were performed within “ParticipantAFQ” using the Vistasoft software package (https://github.com/vistalab/vistasoft). The tractography procedure used 25,000 seed points to generate streamlines through major white matter tracts. DTI metrics, including FA, MD, RD, and AD, were calculated for each tract. FA reflects the directionality of water diffusion and is often associated with fiber integrity; MD measures the overall magnitude of diffusion, indicating tissue density; RD reflects diffusion perpendicular to the main fiber direction, often used to assess myelination; and AD captures diffusion along the main fiber direction, providing insight into axonal integrity.

Tract diffusion profiles were extracted using the “ParticipantAFQ.export(‘profiles’)” command, generating structured arrays of tensor-based metrics for 28 tracts which were segmented into 100 equidistant nodes per group. Our analysis focused on four DTI metrics—FA, MD, AD, and RD—across 100 nodes in eight language-related tracts: the left and right AF, ILF, SLF, and IFOF.

### Lateralization index analysis

The LI for each DTI metric was calculated using the formula: (R – L)/(R + L). A positive LI reflects greater values in the right hemisphere relative to the left, whereas a negative LI reflects greater values in the left hemisphere. One-sample t-tests were conducted in each group (ASD and TD) to assess whether the mean LI for each tract differed significantly from zero. Multiple-comparison correction was applied using 5,000-permutation testing (*p* < 0.05). For FA, which generally reflects microstructural integrity, significant positive *t*-values (corrected *p* < 0.05) indicate rightward lateralization, and significant negative *t*-values indicate leftward lateralization. For MD, RD, and AD, although higher values reflect greater diffusivity rather than greater integrity, the interpretation of the LI itself remains consistent: positive LI values indicate higher diffusivity in the right hemisphere, and negative values indicate higher diffusivity in the left hemisphere. Accordingly, significant negative *t*-values for these diffusivity metrics correspond to rightward lateralization, whereas significant positive *t*-values correspond to leftward lateralization.

To compare the LI between groups for each DTI metric and tract, liner regression models controlling for age and sex were conducted. Next, we conducted a regression analysis between the LI and language scores within each group using a linear regression model: LI ~ language scores + age + sex, controlling for age and sex. To evaluate whether the associations between LI and language scores differed between groups, we conducted a regression analysis that included the interaction term between group and language scores: LI ~ group * language scores + age + sex. Statistical significance was determined using a permutation-based multiple comparison correction with 5,000 permutations (*p* < 0.05).

### Group comparisons in DTI metrics

We compared each DTI metric between the TD and ASD groups at both the tract-wise (mean value across 100 nodes) and point-wise levels using a linear regression model: DTI metric ~ group + age + sex, where group was the independent variable, age and sex as covariates. Statistical significance at the tract-wise level was determined using the permutation-based multiple comparison correction with 5,000 permutations (*p* < 0.05). For the point-wise analysis, consistent with previous research (26), only significant group differences (*p* < 0.05, permutation-based correction) observed across more than three consecutive nodes were considered significant.

### Relationships between DTI metrics and language scores in both TD and ASD groups

We examined the relationships between each DTI metric and the GDS language scores separately for the ASD and TD groups at both the tract-wise (mean value across 100 nodes) and point-wise levels (mean value across more than three adjacent nodes showing significant group differences). The relationships were analyzed using the linear regression model: DTI metric ~ language scores + age + sex, controlling for age and sex. To evaluate whether the strength of these associations differed between groups, we conducted a regression analysis that included the interaction term between group and language scores: DTI metric ~ group * language scores + age + sex. Multiple comparisons were corrected using permutation testing with 5,000 permutations (*p* < 0.05).

### Relationships between DTI metrics and ASD symptom severity in the ASD group

To investigate the relationship between DTI metrics and symptom severity of ASD, we conducted a regression analysis between each DTI metric and total ADOS scores within the ASD group. This analysis was performed at both the tract-wise and point-wise levels (mean value across more than three adjacent nodes showing significant group differences) using a similar linear regression model: DTI metric ~ ADOS total scores + age + sex, controlling for age and sex. Permutation tests were applied to correct for multiple comparisons with 5,000 permutations (*p* < 0.05).

## Results

### Participant characteristics and group comparisons

Participants in the TD and ASD groups were matched on age and sex (*p*s > 0.05). Children with ASD exhibited significantly higher ABC scores than TD peers (*t*_(48)_ = -13.93, *p* < 0.001). However, children with ASD had lower GDS scores in five domains and total scores than TD children (*p*s < 0.001). Detailed demographic, clinical, and behavioral characteristics of both groups are provided in **Table 1**.

**Table 1:** Demographic, clinical, and behavioral characteristics of the TD and ASD groups.

Characteristics	TD		ASD		*t* value	*p* value
	(n = 22)		(n = 28)			
	Mean ± SD	Range	Mean ± SD	Range		
Age (years)	2.97 ± 1.31	1.5-5.81	3.24 ± 1.26	1.67-6.07	-0.73	0.467
Sex (M/F)	17/5		24/4		1.74^a^	0.48
ABC	23.59 ± 7.7	8-40	70.25 ± 14.13	53-107	-13.93	< 0.001
ADOS						
SA	–	–	12.75 ± 3.69	7-22	–	–
RRB	–	–	1.18 ± 0.77	0-3	–	–
Total	–	–	13.93 ± 4.18	8-24	–	–
GDS						
Language	93.51 ± 4.03	83.3-101	46.8 ± 11.9	23.6-70.7	17.60	< 0.001
Personal-social	94.07 ± 4.01	83.3-103	55.59 ± 9.15	32.4-70.2	18.36	< 0.001
Gross motor	98.38 ± 5.5	83-107	76.17 ± 7.07	64.1-89.3	12.13	< 0.001
Fine motor	96.43 ± 4.82	86-105	68.72 ± 9.61	45.9-84.2	12.34	< 0.001
Adaptive behavior	95.44 ± 4.09	88-105	60.54 ± 11.39	38.6-73.7	13.67	< 0.001
Total	95.08 ± 3.85	86.1-100.8	61.58 ± 7.99	43.5-73.6	18.06	< 0.001

TD, typical development; ASD, autistic spectrum disorder. ABC, Autism Behavior Checklist; ADOS, Autism Diagnostic Observation Schedule; SA, Social Affect; RRB, Restricted and Repetitive Behaviors; GDS, Gesell Developmental Schedules. ^a^*Fisher*’s exact test.

### Results of within- and between-group lateralization analyses

**Figure 1:**
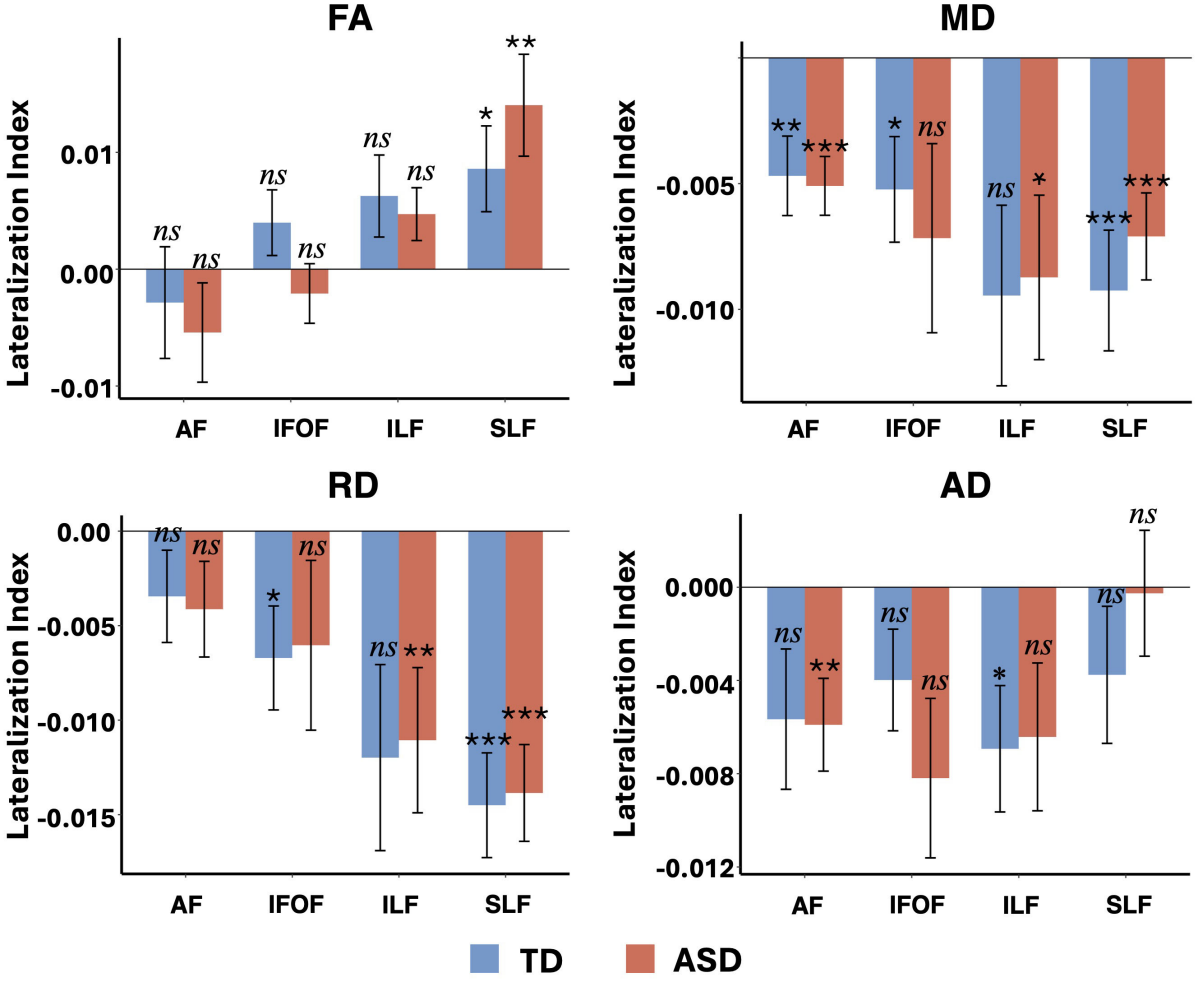
The lateralization index (LI) for each DTI metric (FA, MD, RD, and AD) in the four white matter tracts for both TD and ASD groups. Significant results from the one-sample *t*-tests are indicated by asterisks. *p < 0.05; **p < 0.01; ***p < 0.001; *ns*, not significant. Error bars represent standard errors. Positive LI denotes rightward lateralization, while negative LI indicates leftward lateralization. AF, arcuate fasciculus; IFOF, inferior fronto-occipital fasciculus; ILF, inferior longitudinal fasciculus; SLF, superior longitudinal fasciculus. FA, fractional anisotropy; MD, mean diffusivity; RD, radial diffusivity; AD, axial diffusivity.

As shown in **Table 2**; **Figure 1**, there were significant lateralization patterns across DTI metrics after permutation-based correction. For FA, both TD and ASD groups showed significant rightward lateralization in the SLF (TD: *t*_(21)_ = 2.34, *p* = 0.029; ASD: *t*_(27)_ = 3.22, *p* = 0.003). For MD, significant lateralization was observed in the AF and SLF for both groups (all *p*s < 0.05); additionally, the ASD group showed rightward significant lateralization in the ILF (*t*_(27)_ = -2.67, *p* = 0.013), whereas the TD group showed significant rightward lateralization in the IFOF (*t*_(21)_ = -2.49, *p* = 0.021). For RD, significant lateralization was found in the IFOF and SLF for the TD group, and in the ILF and SLF for the ASD group (*p*s < 0.05). For AD, the TD group showed significant rightward lateralization in the ILF (*t*_(21)_ = -2.55, *p* = 0.018), whereas the ASD group showed significant rightward lateralization in the AF (*t*_(27)_ = -2.97, *p* = 0.006). However, the between-group lateralization analyses using linear regression models did not show any significant differences between the TD and ASD groups after permutation-based corrections (**Supplementary Table S1**).

**Table 2:** Mean and standard deviation of LI for each metric within the four tracts for each group, along with the corresponding *t* and *p* values from the one-sample *t*-tests.

DTI metric	Fiber tract	TD				ASD			
		Mean	SD	*t*-value	*P*-value	Mean	SD	t-value	*P*-value
FA	AF	-0.0029	0.0224	-0.60	0.556	-0.0054	0.0225	-1.27	0.214
	IFOF	0.0040	0.0132	1.42	0.171	-0.0021	0.0135	-0.82	0.421
	ILF	0.0063	0.0165	1.78	0.089	0.0047	0.0117	2.09	0.047
	SLF	0.0086	0.0172	2.34	**0.029**	0.0140	0.0231	3.22	**0.003**
MD	AF	-0.0047	0.0074	-2.97	**0.007**	-0.0051	0.0062	-4.34	**< 0.001**
	IFOF	-0.0052	0.0098	-2.49	**0.021**	-0.0072	0.0199	-1.91	0.067
	ILF	-0.0095	0.0168	-2.64	0.015	-0.0087	0.0170	-2.67	**0.013**
	SLF	-0.0092	0.0113	-3.85	**< 0.001**	-0.0071	0.0091	-4.11	**< 0.001**
RD	AF	-0.0034	0.0114	-1.41	0.173	-0.0041	0.0134	-1.63	0.114
	IFOF	-0.0067	0.0129	-2.44	**0.024**	-0.0060	0.0237	-1.34	0.190
	ILF	-0.0120	0.0231	-2.43	0.024	-0.0111	0.0199	-2.88	**0.008**
	SLF	-0.0145	0.0130	-5.24	**< 0.001**	-0.0139	0.0135	-5.42	**< 0.001**
AD	AF	-0.0057	0.0141	-1.88	0.074	-0.0059	0.0105	-2.97	**0.006**
	IFOF	-0.0040	0.0102	-1.83	0.082	-0.0082	0.0181	-2.40	0.024
	ILF	-0.0069	0.0127	-2.55	**0.018**	-0.0064	0.0165	-2.03	0.053
	SLF	-0.0038	0.0138	-1.28	0.215	-0.0003	0.0143	-0.10	0.924

LI, lateralization index; AF, arcuate fasciculus; IFOF, inferior fronto-occipital fasciculus; ILF, inferior longitudinal fasciculus; SLF, superior longitudinal fasciculus. FA, fractional anisotropy; MD, mean diffusivity; RD, radial diffusivity; AD, axial diffusivity. Bolded *p*-values indicate significant lateralization indices that survived permutation-based correction for multiple comparisons.

### Relationships between lateralization index and language abilities

**Figure 2:**
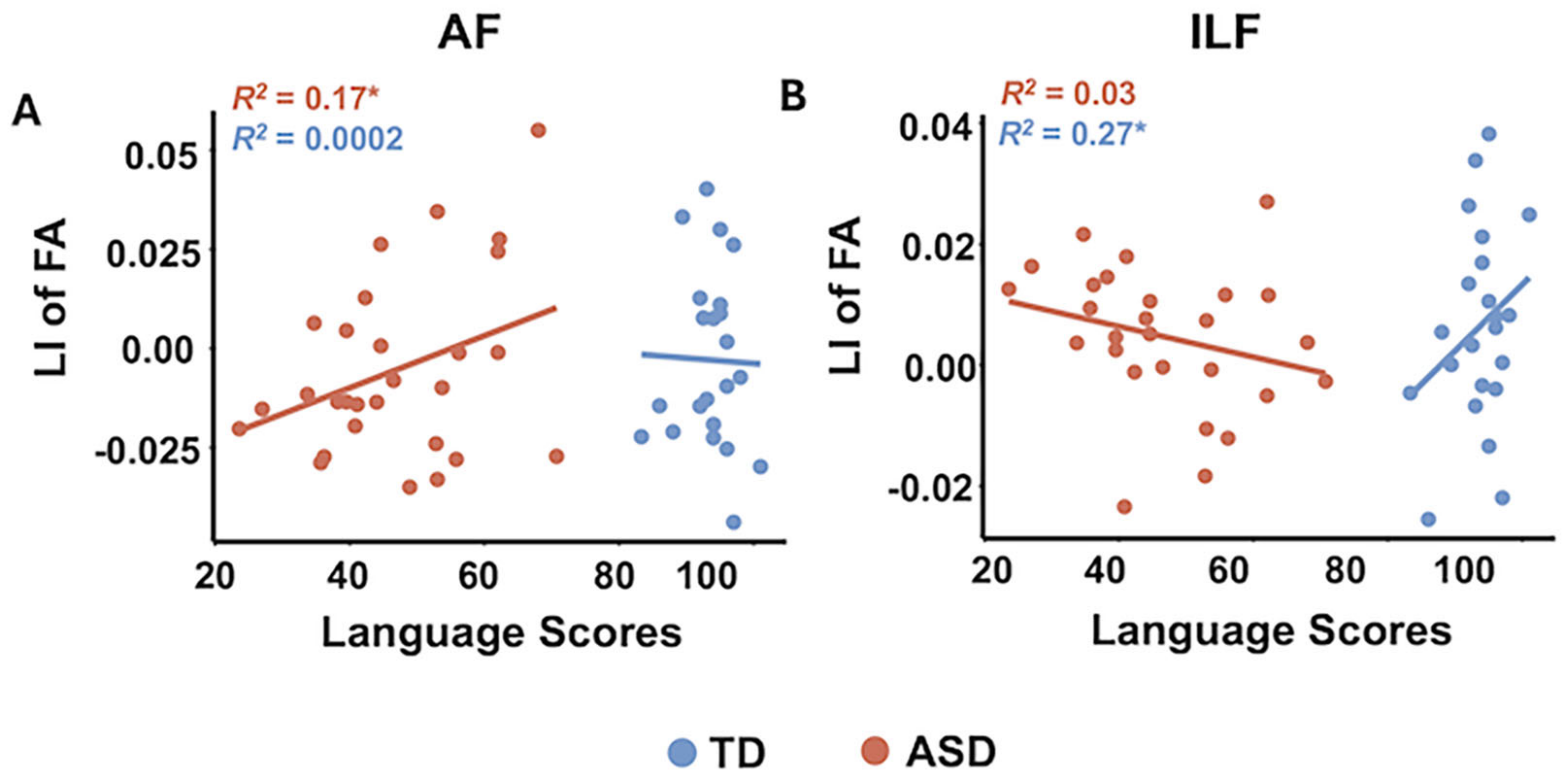
Scatterplots presenting significant associations between the LI of FA in the Arcuate Fasciculus (AF) and language scores in the ASD group **(A)**, and significant associations between the LI of FA in the inferior longitudinal fasciculus (ILF) and language scores in the TD group **(B)**. The *R^2^* values were calculated from partial correlation analysis, controlling for age and sex. Significant results corrected for multiple corrections using permutation tests with 5,000 iterations (*p* < 0.05) are indicated by asterisks. Abbreviations: LI, lateralization index; FA, fractional anisotropy.

As shown in **Figure 2**, a significantly positive association between the LI of FA and language scores was observed in the AF (*β* = 0.0008, SE = 0.0004, *t*_(24)_ = 2.2, *p* = 0.037) in the ASD group, and a significantly positive associations between the LI of FA in the ILF and language scores (*β* = 0.002, SE = 0.0009, *t*_(18)_ = 2.59, *p* = 0.019) was found in the TD group. There was also a significant group × language interaction for the LI of FA in the ILF (*β* = 0.002, SE = 0.0008, *t*_(44)_ = 2.34, *p* = 0.02). No other significant associations were observed in the TD or ASD group after correcting for multiple comparisons.

### Group differences in DTI metrics

**Figure 3:**
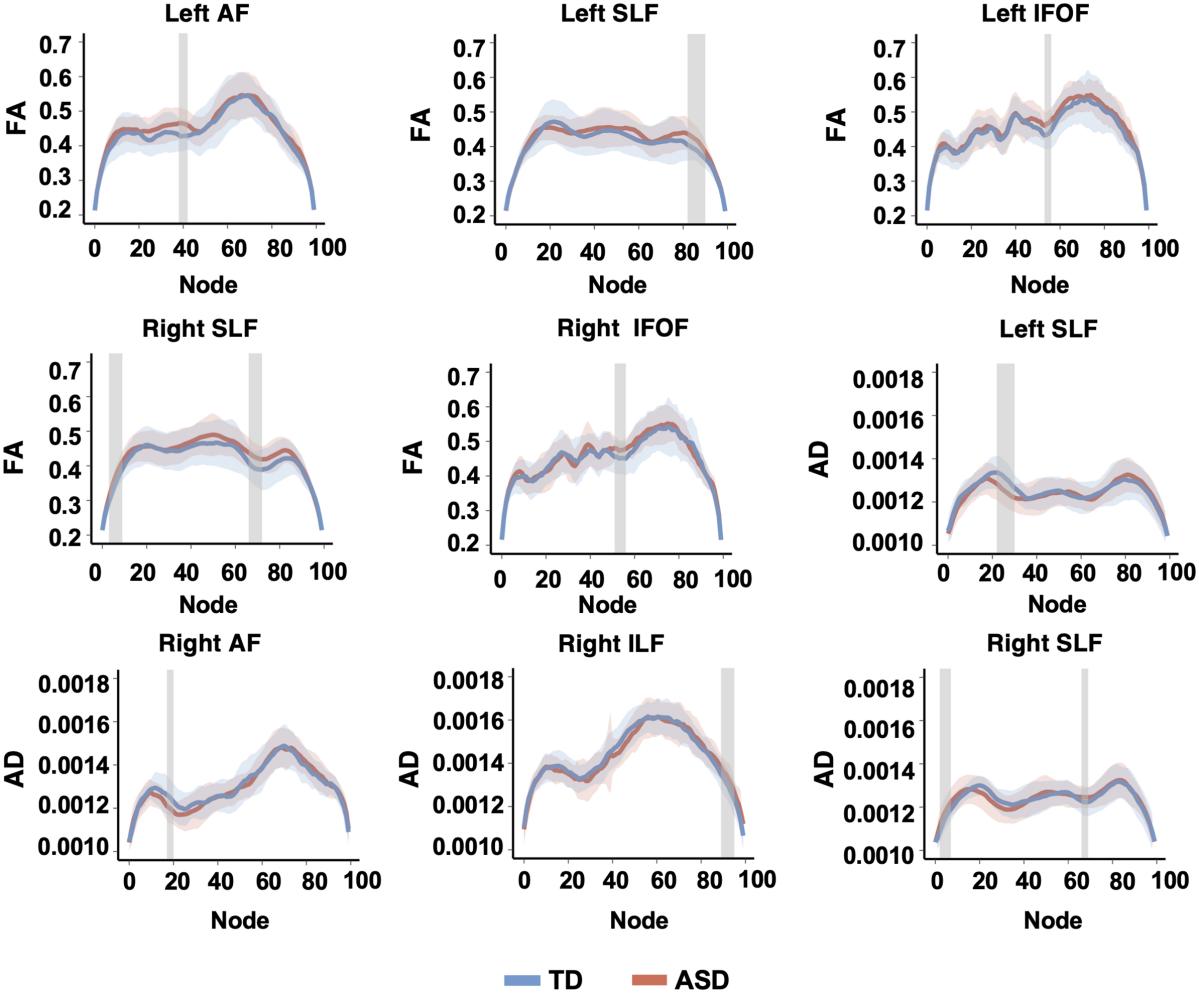
Curve plots depict group differences in white matter fiber tracts at the point-wise level for each DTI metric between TD and ASD children. Solid lines represent mean values, and shaded regions represent standard deviations at each node for each group. Grey bars mark fiber node locations with significant differences, defined as segments with more than three consecutive nodes at *p* < 0.05 after permutation-based corrections. No additional correction for multiple comparisons was applied. AF, arcuate fasciculus; SLF, superior longitudinal fasciculus; IFOF, inferior fronto-occipital fasciculus; ILF, inferior longitudinal fasciculus; FA, fractional anisotropy; AD, axial diffusivity.

At the tract-wise level, no significant group differences were found for any of the DTI metrics (**Supplementary Figure S1**). However, significant differences emerged at the point-wise level (**Figure 3**), where more than three consecutive significant nodes (*p* < 0.05, permutation corrected) were observed, following the approach used in previous research (26). No additional corrections for multiple comparisons were performed. Notable findings included significantly higher FA values in the ASD group compared to TD controls in specific regions: left AF (nodes 38–42), left SLF (nodes 82–90), left IFOF (nodes 53–56), right SLF (nodes 3–9, 66–72), and right IFOF (nodes 51–56). The ASD group showed reduced AD values in the left SLF (nodes 22–30) and right AF (nodes 17–20), but higher AD values in the right ILF (nodes 89–95) and right SLF (nodes 2–7, 66–69) compared to the TD group.

### Significant relationships between DTI metrics and language abilities in the ASD group

**Figure 4:**
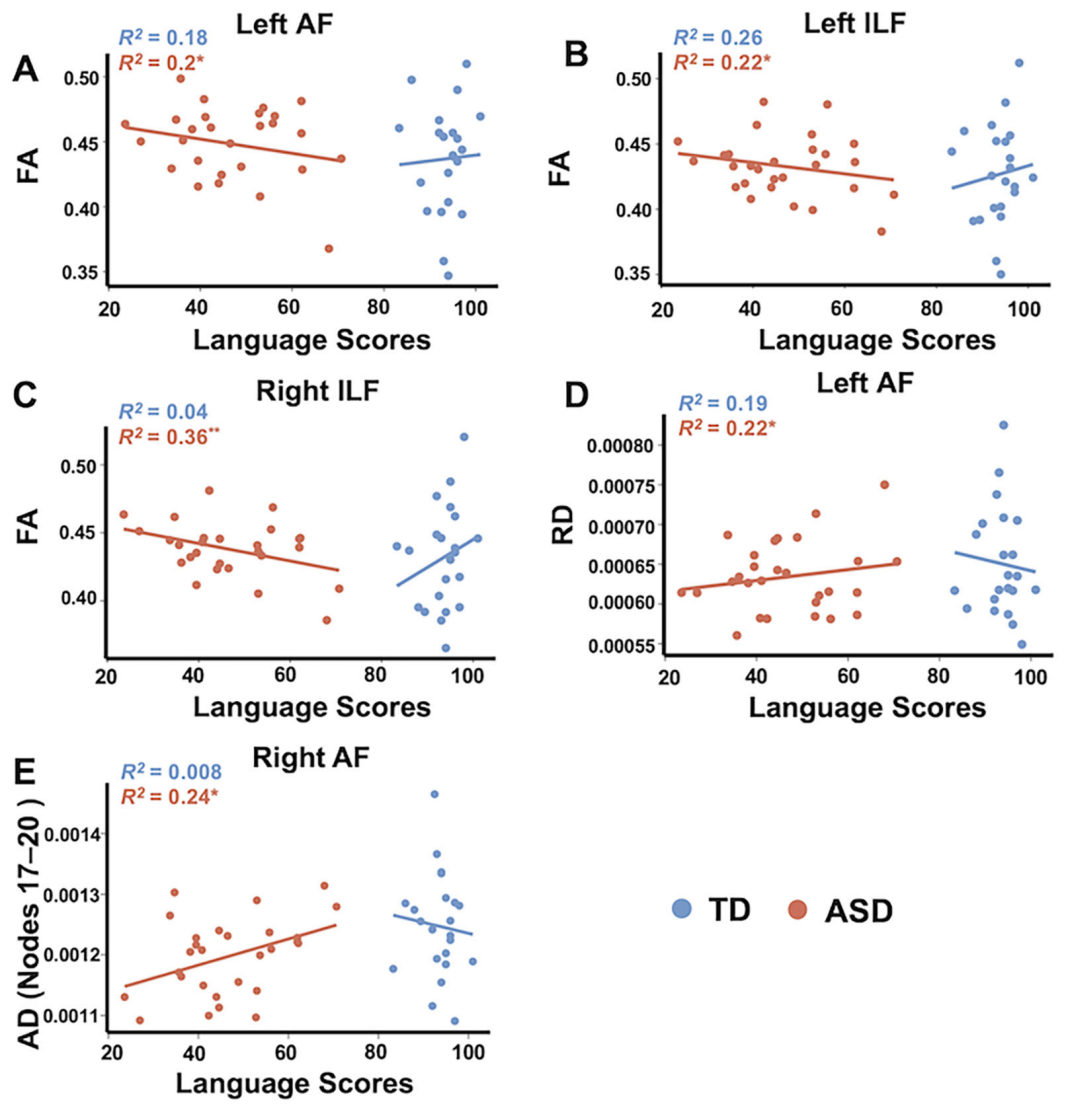
Scatterplots displaying significant associations between mean DTI metrics and language scores in the ASD group in FA of the left AF **(A)**, FA of the left ILF **(B)**, FA of the right ILF **(C)**, RD of the left AF **(D)**. There were also significant associations between mean DTI metrics across nodes 17–20 and language scores in the ASD group **(E)**. The asterisk indicates significant correction after permutation correction. *p < 0.05. The *R^2^* values were calculated from partial correlation analysis, controlling for age and sex. ILF, inferior longitudinal fasciculus; AF, arcuate fasciculus; FA, fractional anisotropy; RD, radial diffusivity; AD, axial diffusivity.

The ASD group demonstrated significantly negative associations between mean FA and language scores in the left AF (*β* = -0.001, SE = 0.0004, *t*_(24)_ = -2.48, *p* = 0.021; **Figure 4A**), left ILF (*β* = -0.0008, SE = 0.0003, *t*_(24)_ = -2.62, *p* = 0.015; **Figure 4B**), and right ILF (*β* = -0.0009, SE = 0.0003, *t*_(24)_ = -3.59, *p* = 0.002; **Figure 4C**), controlling for age and sex. Additionally, the ASD group showed a significant positive association between mean RD in the left AF and language scores (*β* = 1.46e-06, SE = 5.6e-07, *t*_(24)_ = 2.61, *p* = 0.015; **Figure 4D**). Following permutation-based correction, all other DTI metrics showed no significant association in either the ASD or TD group, nor any significant group × language interactions (all *p*s > 0.05).

Among associations between mean DTI metrics at nodes with significant point-wise group differences and language scores across various white matter tracts, the only significant finding was a positive association between mean AD in the right AF (nodes 17–20) and language scores in the ASD group (*β* = 2.64e-06, SE = 9.7e-07, *t*_(24)_ = 2.73, *p* = 0.012; **Figure 4E**). No additional node-level associations survived permutation-based correction in either the ASD or TD group. Moreover, no significant group × language scores interaction was observed (*p* > 0.05).

### Significant relationships between DTI metrics and symptom severity in the ASD group

**Figure 5:**
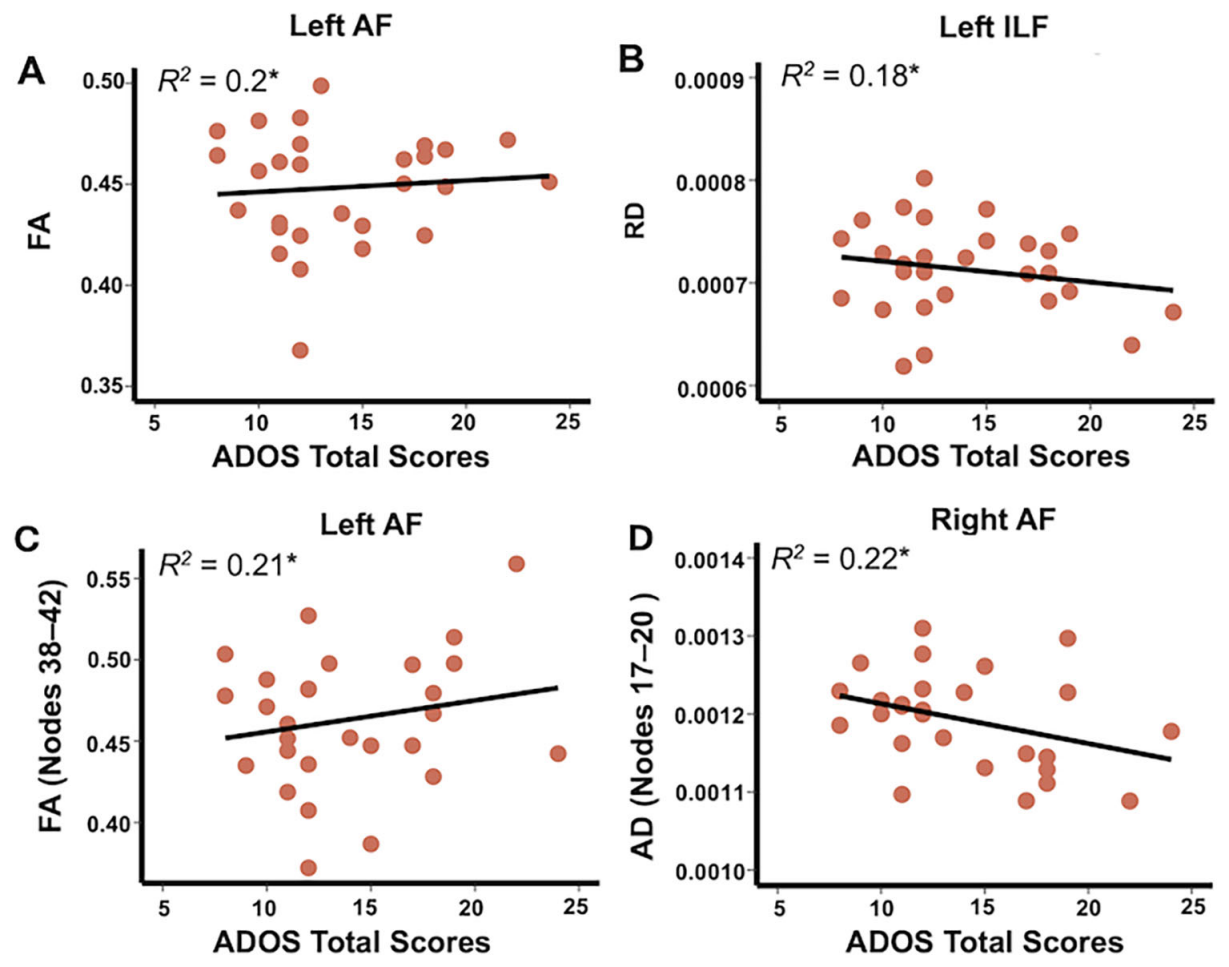
Scatterplots illustrating significant associations between mean DTI metrics and symptom severity of ASD (ADOS total scores) in FA of the left AF **(A)**, RD of the left ILF **(B)**, and between mean DTI metrics at nodes with significant group differences and symptom severity of ASD in FA of the left AF (nodes 38–42; **C**) and AD of the right AF (nodes 17–20; **D**). The asterisk indicates the significant associations following permutation test correction (5,000 permutations, *p* < 0.05). AF, arcuate fasciculus; FA, fractional anisotropy; RD, radial diffusivity; AD, axial diffusivity.

Significant associations between the mean DTI metrics and ADOS total scores were observed in the ASD group after correcting for multiple comparisons (**Figure 5**). Significantly positive associations between mean FA and ADOS total scores were observed in the left AF (*β* = 0.003, SE = 0.001, *t*_(24)_ = 2.43, *p* = 0.023) and a negative association between mean RD and ADOS total scores was noted in the left ILF (*β* = -4.7e-06, SE = 2.02e-06, *t*_(24)_ = -2.3, *p* = 0.028). No other significant associations were found between mean AD and ADOS total scores in any tract after correcting for multiple comparisons using the permutation-based corrections.

Additionally, a significantly positive association was found between ADOS total scores and mean FA in the left AF (nodes 38–42) (*β* = 0.005, SE = 0.002, *t*_(24)_ = 2.56, *p* = 0.017), and conversely, a significantly negative association was observed between ADOS total scores and mean AD in the right AF (nodes 17–20) (*β* = -8.08e-06, SE = 3.1e-06, *t*_(24)_ = -2.61, *p* = 0.015).

## Discussion

Our study used DTI and AFQ to examine white matter in young children with ASD and TD controls. While no significant group differences in lateralization were found, both groups showed distinct lateralization patterns and their associations with language abilities. Point-wise analyses revealed localized differences in FA, AD, RD, and MD, despite no tract-level group differences. DTI metrics were also linked to language abilities and, in the ASD group, to symptom severity. These results highlight the role of white matter lateralization and microstructure in language function and their potential link to early language deficits and symptom severity in ASD.

Notably, we observed significant associations between white matter integrity and language abilities in the ASD group. Specifically, FA in the bilateral ILF and the left AF was negatively correlated with language scores in children with ASD, whereas no such associations were observed in the TD group. These results support our hypothesis and underscore the distinct relationship patterns between white matter integrity and language abilities in ASD compared with TD. In TD children, more organized white matter—characterized by higher FA and lower RD—is generally associated with better language performance (27, 28). Conversely, in children with ASD, higher FA and lower RD may indicate atypical neural development that does not correlate with better language outcomes. This suggests that the neurodevelopmental processes underlying language acquisition in ASD may involve compensatory mechanisms or alternative neural pathways.

Within the ASD group, significant associations were identified between DTI metrics and the severity of ASD symptom. Specifically, higher FA in the left AF and left SLF was associated with greater ASD severity. This finding contradicts previous research, such as Zhang et al. (29), which linked decreased FA in the AF to increased language deficits, and Im et al. (5), which found lower FA in the SLF correlating with poorer social interaction scores measured by the Autism Diagnostic Interview-Revised in adolescents. Additionally, we observed that lower MD and RD in the left AF, left ILF, and bilateral SLF were associated with increased ASD severity. This finding is inconsistent with Li et al. (18), which did not report such associations between DTI metrics and ASD symptom severity. These discrepancies may be due to differences in participant characteristics and age, as previous studies often focused on older individuals or children with language regression (5, 18; L. 29). At the point-wise level, we found that higher FA in the left AF and lower AD in the right AF were significantly associated with greater ASD severity, further refining our observations. These findings underscore the complexity of white matter alterations in ASD, with distinct tracts showing varying patterns of association with symptom severity, which may depend on specific characteristics and developmental stages.

Contrary to our hypothesis, no significant group differences in mean FA, MD, AD, or RD were observed at the tract-wise level, suggesting similar white matter integrity between ASD and TD children. This finding is consistent with several previous studies in young children with ASD, which have reported inconsistent or null group differences in white matter organization (15, 30, 31). These results suggest that widespread white matter alterations may not be a robust or early-emerging feature of ASD. However, point-wise analyses uncovered significant group differences in specific nodes. Compared to TD children, children with ASD exhibited higher FA in nodes of the left AF, and bilateral SLF and IFOF. The higher FA in these tracts may suggest increased axonal density, greater coherence of fiber orientation, or reduced complexity of crossing fibers. In contrast, children with ASD exhibited lower AD values in nodes of the left SLF and right AF, which may indicate alterations in axonal microstructure or reduced longitudinal diffusivity. Meanwhile, increased AD in nodes of the right ILF and right SLF may suggest enhanced myelination or more organized fiber architecture in these regions. These findings underscore the intricate and varied nature of white matter alterations in ASD, which are manifested as both increases and decreases in DTI metrics across different brain tracts.

Both groups exhibited notable patterns of hemispheric lateralization in white matter tracts. Specifically, both ASD and TD children exhibited significant rightward lateralization in the SLF for FA, as well as rightward lateralization in the SLF and AF for MD, and in the SLF for RD. Beyond these shared patterns, group-specific effects also emerged. The ASD group demonstrated significant rightward lateralization in the ILF for MD and RD, and in the AF for AD. In contrast, the TD group showed significant rightward lateralization in the IFOF for MD and RD, and in the ILF for AD. The overall rightward lateralization observed in language-related tracts in both groups is consistent with previous findings of greater right-hemisphere white matter integrity—characterized by higher FA and lower MD and RD—relative to the left (32). These findings also converge with earlier reports of rightward asymmetry in the IFOF and AF (33). Notably, the rightward lateralization observed here contrasts with the left-lateralization of the AF and other language-related pathways commonly reported in older children and adults (e.g., 10, 33). One likely explanation for the absence of pronounced leftward asymmetry in our sample is developmental: our participants are very young (1.5–6 years), a period during which hemispheric specialization for language is still emerging. Because only a limited number of studies (e.g., 34) have examined structural asymmetry in very young children, future work is needed to clarify how these lateralization patterns evolve across early development.

Despite the asymmetry in DTI metrics, there were no significant differences in lateralization between the ASD and TD groups, indicating that white matter lateralization patterns in the ASD group did not differ substantially from those in TD children at this stage of early development. This contrasts with prior studies reporting reduced left lateralization in ASD compared to TD children in tracts like the AF (10), as well as in the SLF, ILF, and IFOF (32, 34). These discrepancies may reflect sample-specific factors, such as age range and demographics, suggesting that lateralization patterns can vary across studies. This underscores the need for diverse samples and consistent methods. Further research should clarify how white matter lateralization develops in ASD across different ages and populations.

Our analysis revealed significant associations between white matter lateralization and language abilities in both ASD and TD children. In children with ASD, a more pronounced right-lateralized pattern in the AF, as reflected by higher FA, was associated with better language abilities. This finding aligns with the known role of the AF in supporting key language functions such as speech production and comprehension, particularly in its connections between Broca’s and Wernicke’s areas (35, 36). The enhanced right-lateralization in ASD may reflect compensatory neural mechanisms that support language processing in the right hemisphere (37). In contrast, TD children exhibited a positive association between a more right-lateralized pattern in the ILF and better language abilities. The stronger right-lateralization in TD children could indicate the importance of right hemisphere pathways in supporting more integrative, higher-order language functions, such as contextual understanding and word retrieval, in TD children. These results suggest that FA might be the most sensitive metric for capturing relevant microstructural properties of white matter tracts that are relevant for language function. These group-specific patterns highlight differing neurodevelopmental trajectories and hemispheric specialization in ASD and TD children. Longitudinal studies are needed to track how these patterns evolve with development.

### Limitations

Several limitations should be considered when interpreting these findings. Firstly, although permutation testing was applied to correct for multiple comparisons, the point-wise analyses remain exploratory and rely on relatively liberal statistical thresholds. As such, the point-wise results should be interpreted with caution. Secondly, while our point-wise analyses revealed detailed white matter changes in ASD, localizing the exact anatomical coordinates of these affected nodes remains challenging. This limitation, common in AFQ studies (38–40), arises from the reliance on fiber segmentation in individual native space. Thirdly, AFQ only analyzes the central portion of each fiber tract by clipping streamlines to the segment between the two defining ROIs, thereby focusing solely on the deep white matter “stem” of the tract (17). As a result, peripheral or branching segments are not evaluated, which may limit the ability to capture the full spatial extent of tract-specific alterations. Fourth, the DTI model cannot resolve crossing or branching fibers within a single voxel, which may lead to artificially reduced FA and altered MD, AD, or RD values in regions with complex white-matter architecture. Consequently, tract-based metrics—particularly in intersecting pathways such as the SLF, AF, and ILF—should be interpreted with caution. Finally, ASD symptoms may emerge later in development, particularly in children as young as those included in our control group. Future studies should incorporate longitudinal follow-up to determine whether any control participants later received an ASD diagnosis.

## Conclusion

Our findings add to the growing literature on white matter development in young children with ASD. Although no tract-level differences emerged, localized increases in FA, MD, and AD—and decreases in RD and AD—were observed in the ASD group. Notably, AD in the right AF positively correlated with language scores. These results underscore the value of examining specific tract nodes and suggest that subtle white matter alterations, rather than gross alternations, may contribute to early language and social deficits in ASD. Longitudinal studies are needed to clarify developmental trajectories and identify early biomarkers and intervention targets.

## Data Availability

The data used for the analyses reported in this article are available at: https://github.com/Yaqiongxiao/asdDTI.language.
