# Supplementary material for: Altered white matter connectivity is linked to language abilities in children with autism spectrum disorder: An automated fiber quantification study
Source: Front Psychiatry. 2026 Jan 20;16:1731647. doi: 10.3389/fpsyt.2025.1731647 (PMC12864472; doi:10.3389/fpsyt.2025.1731647)
Supplement: Supplementary file 1 [file Table1.docx]

**Supplementary materials**

**Table S1.** Mean LI differences (TD vs. ASD) for each DTI metric in the four tracts, with corresponding *F*- and *p*-values from the regression analysis.

| **DTI metric** | **Fiber tract** | **TD vs. ASD** | | |
| --- | --- | --- | --- | --- |
|  |  | **Difference** | ***F*-value** | ***p*-value** |
| **FA** | **AF** | 0.0026 | 0.1600 | 0.6910 |
|  | **IFOF** | 0.0061 | 2.5987 | 0.1138 |
|  | **ILF** | 0.0016 | 0.1622 | 0.6890 |
|  | **SLF** | -0.0054 | 0.8471 | 0.3622 |
| **MD** | **AF** | -0.0061 | -1.599 | 0.117 |
|  | **IFOF** | -0.0019 | -0.450 | 0.655 |
|  | **ILF** | 0.0007 | 0.127 | 0.899 |
|  | **SLF** | -0.0042 | -1.041 | 0.304 |
| **RD** | **AF** | -0.0016 | -0.372 | 0.712 |
|  | **IFOF** | 0.0007 | 0.149 | 0.882 |
|  | **ILF** | 0.0009 | 0.146 | 0.884 |
|  | **SLF** | 0.0005 | 0.123 | 0.903 |
| **AD** | **AF** | 0.0054 | 0.954 | 0.345 |
|  | **IFOF** | 0.0021 | 0.725 | 0.472 |
|  | **ILF** | 0.0006 | 0.170 | 0.865 |
|  | **SLF** | 0.0035 | 0.877 | 0.385 |

Abbreviations: LI, lateralization index; FA, fractional anisotropy; MD, mean diffusivity; RD, radial diffusivity; AD, axial diffusivity; AF, arcuate fasciculus; IFOF, inferior fronto-occipital fasciculus; ILF, inferior longitudinal fasciculus; SLF, superior longitudinal fasciculus.

**
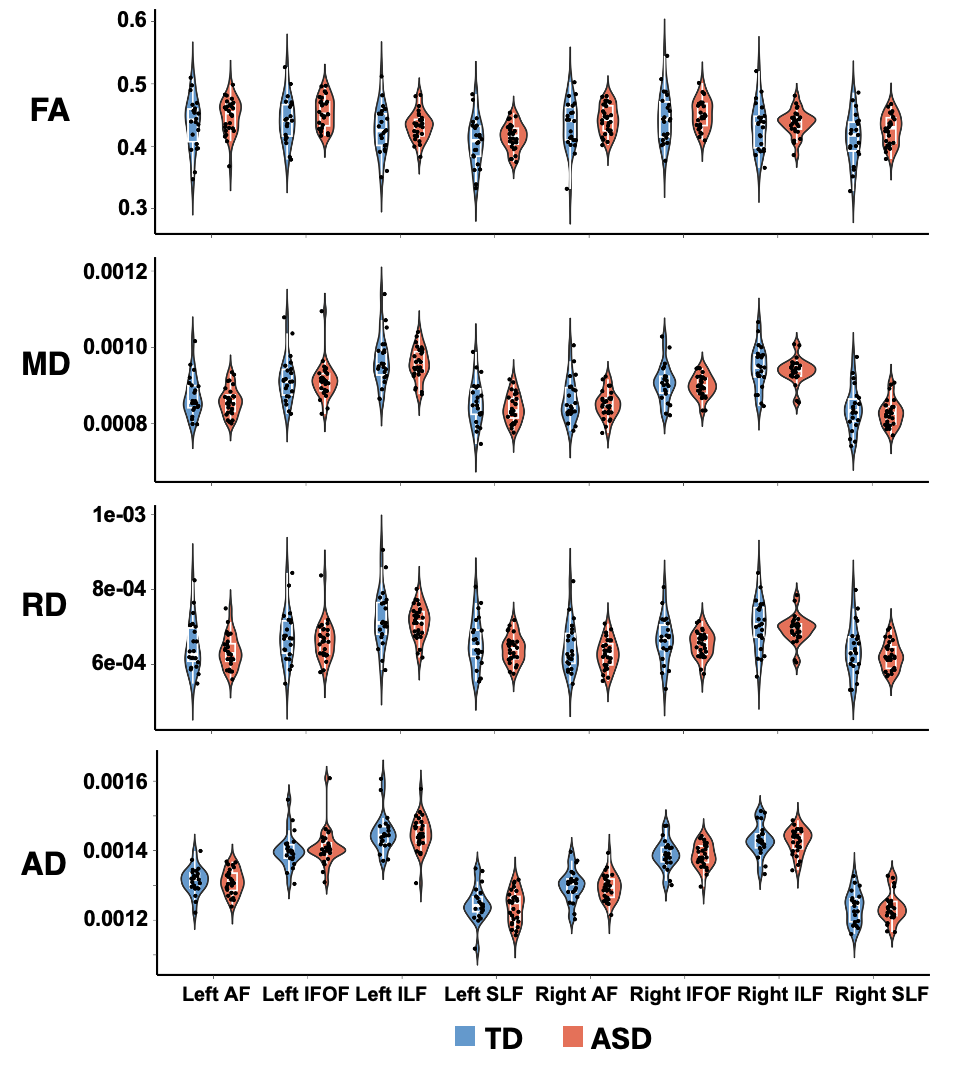
Figure S1.** Grouped violin plots presenting group comparisons of the mean of each DTI metric at the tract-wise level between ASD and TD children. None of the differences between groups are significant (*p*s > 0.05). Abbreviations: AF, arcuate fasciculus; IFOF, inferior fronto-occipital fasciculus; ILF, inferior longitudinal fasciculus; SLF, superior longitudinal fasciculus. FA, fractional anisotropy; MD, mean diffusivity; RD, radial diffusivity; AD, axial diffusivity.
